# Single-cell Raman spectroscopy and synthetic microbiology power microbial-driven extraterrestrial domestic wastewater treatment

**DOI:** 10.1128/msystems.00596-26

**Published:** 2026-06-23

**Authors:** Xu Wang, Haonan Fan, Liangchang Zhang, Yuhan Ge, Cancan Jiang, Xuliang Zhuang

**Affiliations:** 1 Research Center for Eco-Environmental Sciences, Chinese Academy of Sciences 26442, Beijing, China; 2 College of Resources and Environment, University of Chinese Academy of Sciences 617093, Beijing, China; 3 State Key Laboratory of Space Medicine, China Astronaut Research and Training Center 56708, Beijing, China; 4 Division of Life Sciences and Medicine, School of Life Sciences, University of Science and Technology of China 594406, Hefei, Anhui, China; 5 State Key Laboratory of Tibetan Plateau Earth System, Environment and Resources (TPESER), Institute of Tibetan Plateau Research, Chinese Academy of Sciences 666030, Beijing, China; University of Illinois Chicago, Chicago, Illinois, USA

**Keywords:** extraterrestrial domestic wastewater, single-cell Raman spectroscopy, short-cut nitrogen removal, synthetic microbial community

## Abstract

The realization of long-term manned space exploration and extraterrestrial habitation hinges on microbial-based extraterrestrial domestic wastewater (EDW) treatment technology to achieve sustainedly closed-loop water recycling. This perspective recaps the challenges and potential of integrating microbial technology as a sustainable and low-energy alternative for treating EDW compared to physicochemical water recovery systems. Of note, traditional microbial technologies are not directly transferable due to EDW’s unique constraints, including high ammonium, low C/N ratio, and multiple stresses. We proposed how synthetic microbiology integrated with single-cell Raman spectroscopy (SCRS) offers a promising approach to engineer stable, efficient microbiomes tailored for EDW treatment. SCRS coupled with stable isotope probing can enable precise identification and isolation of stress-tolerant functional microorganisms at the single-cell level, bypassing lengthy enrichment methods. SCRS can also serve as a real-time monitoring tool for system optimization and early warning, enabling resilient, intelligently monitored biological systems for extraterrestrial water recycling.

## PERSPECTIVE

The ambition for deep-space exploration and the establishment of extraterrestrial habitation necessitates a fundamental advancement in life support technology (1). A cornerstone of this endeavor is the closed-loop recycling of vital resources, with water being the most critical consumable (2). In confined extraterrestrial habitats, the logistical and economic costs of resupplying water from the Earth are prohibitive, making *in situ* water recovery and recycling indispensable for mission viability (3). Treatment of extraterrestrial domestic wastewater (EDW), a composite of humidity condensate, hygiene water, and urine, presents a unique challenge. It is characterized by high ammonium concentrations (500–1,500 mg/L), a low carbon-to-nitrogen (C/N) ratio due to the presence of recalcitrant organic carbon (e.g., urea), low pH (4.5–6.5), and potential multiple stresses from salinity and specific organic compounds (4, 5). Effective nitrogen removal from EDW is, therefore, a significant hurdle.

Current state-of-the-art water recovery systems utilized to treat EDW primarily rely on integrated physicochemical processes like vapor compression distillation, reverse osmosis, and catalytic oxidation (6, 7). While successful, these systems are burdened by high energy consumption, significant mass/volume penalties, susceptibility to fouling and corrosion, and a dependency on periodic resupply of consumables from the Earth. As mission durations extend and crew sizes increase, generating larger volumes of complex waste streams, these limitations will become increasingly acute. Consequently, there is a pressing need for more sustainable, energy-efficient, and self-regenerative wastewater treatment technologies.

Biological treatment, leveraging the catalytic power of self-replicating microorganisms, offers a compelling solution honed over a century of terrestrial application (8). Microbial processes can achieve high treatment efficiency with minimal energy input. Since the 1990s, extraterrestrial exploration agencies have explored biological systems, notably membrane-aerated biofilm reactors (MABRs), for space-based wastewater treatment (9). More recently, experts involved in relevant extraterrestrial projects have emphasized that microbial technologies are key to perfecting future controlled ecological life support systems (CELSS) (10). However, transplanting terrestrial biological processes directly into the extraterrestrial environment is fraught with challenges, primarily due to the unique composition in EDW and the microgravity conditions that affect fluid dynamics and microbial ecology.

This perspective aims to provide a comprehensive overview of the progress and prospects of microbial technologies for EDW treatment within CELSS. We explore the limitations of conventional approaches, examine the promise and pitfalls of shortcut nitrogen removal (SCNR) processes, and delve into how cutting-edge tools like single-cell Raman spectroscopy (SCRS) are facilitating the design and control of synthetic microbial consortia for space applications. Finally, the system integration challenges and future perspectives on regenerative biological life support systems are discussed with the aid of SCRS technology.

## CURRENT STATE AND LIMITATIONS OF CURRENT EDW TREATMENT SYSTEMS

**Fig 1:**
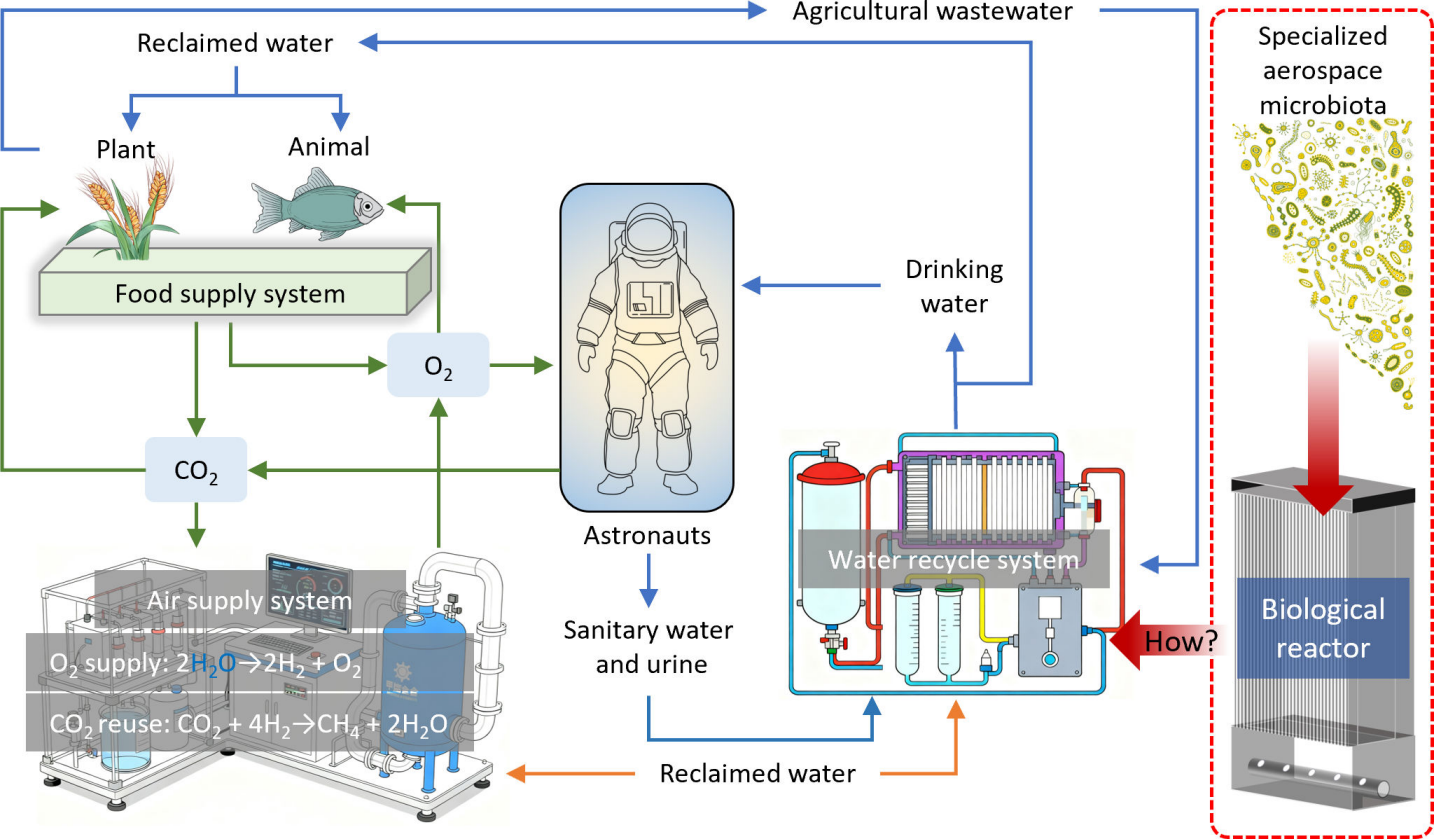
Basic diagram of extraterrestrial CELSS highlighting the closed-loop process of food-air-water cycle and the biological modification of the water recycle system.

An ideal CELSS can achieve sustainable substance recycling through a closed-loop process of food-air-water cycle (Fig. 1). After consuming oxygen and drinking water, astronauts exhale CO_2_ and generate sanitary wastewater and urine. A portion of the CO_2_ is returned to the food supply system, while the remainder enters the air supply system. This air supply system both replenishes oxygen by decomposing water and reutilizes CO_2_ to generate water for subsequent cycles. Simultaneously, agricultural wastewater, along with the sanitary wastewater and urine, enters the water recovery system. After treatment, this water is partly converted into clean water for astronaut consumption and partly regenerated into reclaimed water. This reclaimed water is then recirculated back to both the food supply system and the air supply system. Ultimately, this achieves water recycling and reuse, ensuring life support within the extraterrestrial environment. Therefore, water is the essential medium that drives this entire cycle, and the unit of wastewater treatment is the core motor driving the water recycle (11). The recycling and utilization of water resources is a core technology to ensure astronauts’ long-term stay in extraterrestrial environments.

By combining various physical and chemical methods, it achieves high-efficiency recycling and purification of wastewater such as urine and condensate water (12). The system first employs physical techniques like vapor compression distillation or vacuum membrane distillation to separate pure water vapor from urine (13). Subsequently, the treated water is mixed with condensate water and enters the deep purification stage. This stage integrates reverse osmosis for desalination and relies on chemical technologies such as gas-phase catalytic oxidation or catalytic oxidation reactors to thoroughly decompose organic matter in the wastewater (14). Finally, multilayer filtration and ion exchange are used for fine polishing to remove residual ions and particles (15). The final product is purified water of a quality superior to drinking water standards, significantly reducing dependence on ground supplies. The most representative resource recycling system is the membrane-based Sabatier system, which ingeniously integrates water recovery with propellant production (16). Through catalytic reactions, it converts carbon dioxide exhaled by astronauts and hydrogen recovered from wastewater into water and methane, simultaneously achieving water regeneration and extraterrestrial fuel supply. This exemplifies the circular concept of turning waste into treasure.

However, current physicochemical methods still require periodic resupply of materials from ground bases and can only support long-term space activities for a dozen or so individuals, limiting the establishment of large-scale and complex extraterrestrial habitats. During future deep-space exploration and the construction of lunar bases, which will involve larger crews and more extensive manned activities, significant volumes of high-load wastewater with increasingly complex contaminant profiles will be generated. Such wastewater poses considerable challenges to existing physicochemical treatment approaches. It is imperative to develop self-regenerative, sustainable, and low-consumption wastewater treatment technologies to support long-term manned missions.

## PROGRESS AND PERSISTENT CHALLENGES OF MICROBIAL TECHNOLOGIES FOR EDW TREATMENT

### Limitations of conventional microbial approaches for EDW treatment

Microbial technologies like activated sludge and biofilm reactors are mainstream for terrestrial wastewater treatment, relying on the nitrification-denitrification microbiome for nitrogen removal (17, 18). For space applications, MABR has emerged as the leading biological candidate (19). In an MABR, a gas-permeable membrane supplies oxygen directly to an attached biofilm while substrates (ammonium and organics) diffuse from the bulk liquid. This counter-diffusion configuration is advantageous for simultaneous nitrification and denitrification and is inherently gravity-independent, avoiding the need for gravitational settling (20).

Ground-based research has demonstrated the feasibility of MABRs for EDW treatment. In the 1990s, NASA led the development of numerous membrane bioreactors for treating EDW, aiming to improve the internal recycling of water resources in space stations (9). Pilot-scale systems have achieved >90% total organic carbon (TOC) removal and 50%–65% total nitrogen removal from simulated EDW (19). A China research team tested the effectiveness of MABR in treating simulated EDW during flight phases and domestic wastewater from planetary bases (21). After more than a year of long-term operation, MABR could stably remove almost all TOC, with a total nitrogen removal efficiency of over 65%. Significantly, MABR has been chosen as the specific microbial reactor system for treating EDW (Fig. 1), rather than the more widely used activated sludge reactors on Earth. The main reason is that, in the microgravity environment of space stations, MABR has the advantage of not relying on gravitational sedimentation for liquid-solid separation, thereby accelerating water regeneration.

Despite these promising results, biological EDW treatment has not yet been implemented in orbit. Two major technical bottlenecks persist. First is the low nitrogen removal efficiency. The reported 50%–65% nitrogen removal efficiencies are insufficient for practical application. This inefficiency stems from the low bioavailable C/N ratio (<3) in EDW, which limits heterotrophic denitrification (9, 18, 22). While EDW contains organic carbon (e.g., urea), it is often not readily bioavailable for denitrifiers. Second, system instability and pH sensitivity constrain the activities of nitrogen conversion microbes. Nitrification consumes alkalinity, exacerbating the inherent acidity of EDW and potentially inhibiting microbial activity. Although pH control is possible, it adds operational complexity (23). Therefore, developing MABR technologies capable of efficient, stable nitrogen removal under low C/N and acidic conditions is crucial.

### Promise and instability of short-cut nitrogen removal

Short-cut nitrogen removal (SCNR) processes, such as nitritation/denitritation or partial nitrification and anammox, offer a strategic advantage for low C/N wastewater (24, 25). SCNR halts nitrification at nitrite (NO_2_^−^) by selectively inhibiting nitrite-oxidizing bacteria (NOB), subsequently reducing NO_2_^−^ directly to N_2_ gas (26–28). Therefore, ammonia-oxidizing bacteria (AOB) serve as the core microbial strain that initiates the SCNR process. They achieve this by rapidly oxidizing ammonium (NH_4_^+^) to nitrite (NO_2_^−^), thereby supplying the essential substrate for the subsequent denitrification or anammox phases and driving the entire efficient, low-consumption nitrogen removal pathway. As the initial and rate-limiting step of nitrification, the activity and enrichment level of AOB directly determine the startup efficiency, operational stability, and nitrogen removal performance of the SCNR system. SCNR bypasses the nitrate step, saving approximately 25% aeration energy and 40% organic carbon demand compared to full nitrification-denitrification (29–31), making it theoretically ideal for EDW. However, research works on applying SCNR technology to treat EDW remain scarce.

SCNR is typically established *in situ* from conventional nitrifying-denitrifying communities by applying selective pressures like low dissolved oxygen, high free nitrous acid (FNA), or specific pH (24, 25). However, high-throughput sequencing often reveals a persistent, low-abundance NOB reservoir (0.1%–1%) within these systems. During long-term operation, NOB can adapt or resurge, leading to the breakdown of SCNR performance and reversion to full nitrification (32, 33). This inherent instability poses a significant risk for long-duration space missions where reliability is paramount. Thus, there is an urgent need to construct stable SCNR systems free from NOB resurgence from the outset.

### *De novo* construction of a synthetic SCNR microbiome: theoretical exclusion of NOB for enhanced stability

Synthetic microbial community (SynCom) is an artificially assembled or modified microbial consortium based on the fundamental principles of ecology and synthetic biology (34). It is created by combining two or more distinct microorganisms with known taxonomic status and functional characteristics in a specific ratio under defined conditions. The resulting community is highly efficient, robust, and readily applicable (35). The constituent strains of a SynCom can be wild-type isolates from natural environments or genetically modified microbes (36). Compared to complex, undefined natural communities, SynCom offers predictability, controllability, and the potential for enhanced robustness. Currently, SynComs can provide precise and efficient solutions for complex wastewater treatment (37).

The core challenge in maintaining a stable SCNR microbiome is preserving the core functional strains, AOB and denitrifiers, and selectively inactivating indigenous NOB. However, the indigenous NOB coexisting with AOB is not completely removed via current published *in situ* methods. The SynCom approach may fundamentally circumvent this issue through *de novo*, bottom-up construction of the SCNR microbiome. Core members are selected exclusively based on the SCNR blueprint. This includes specific AOB strains to perform the initial step, paired with compatible denitrifiers that can use nitrite as an electron acceptor. Auxiliary strains for removing other pollutants can be added modularly. Crucially, at the initial design and assembly stage, NOB are categorically excluded from the consortium roster. Since a SynCom is initiated in a sterile or simplified background, the absence of NOB from the inoculum means there is no genomic basis for their activity within the system. This theoretically eliminates the primary internal risk of SCNR destabilization. In fact, the approach of a synthetic SCNR microbiome may be particularly apt for applications in space, primarily because the near-sterile environment of space prevents exogenous microbial contamination from altering the structure of SynCom.

Nevertheless, the theoretical exclusion of NOB does not guarantee permanent stability. Even in a near-sterile space environment, several challenges could compromise NOB-free operation. First, trace amounts of NOB might be introduced via crew-associated microbiota, equipment surfaces, or incompletely sterilized influent. Second, metabolic cross-feeding between AOB and heterotrophs could create micro-niches that favor NOB outgrowth over extended mission durations. Third, adaptive evolution or horizontal gene transfer might confer nitrite-oxidizing capacity to other community members. To address these risks, SCRS can serve as a detection tool for NOB signatures. For example, a reference spectral library can be established by collecting Raman fingerprints of representative NOB strains (e.g., *Nitrospira* and *Nitrobacter*), including characteristic peaks associated with nitrite oxidoreductase and other taxon-specific markers. During operation, SCRS from the bioreactor can be continuously acquired and matched against this library. A detection threshold of NOB abundance can be predefined, and once the threshold is crossed, an alert is triggered. Thus, SCRS not only enables *de novo* construction of NOB-free SynComs but also provides a continuous vigilance mechanism to safeguard long-term SCNR stability.

## SINGLE-CELL RAMAN SPECTROSCOPY: A DISRUPTIVE TOOL FOR SynCom CONSTRUCTION TAILORED FOR EDW TREATMENT

Constructing a SynCom for EDW treatment requires strains that are not only functional efficient but also tolerant to its multiple stresses. Traditional methods for obtaining such strains rely on selective enrichment culturing, which is time-consuming (especially for slow-growing autotrophs like AOB) and often yields mixed cultures rather than pure isolates (38). For instance, acid-tolerant AOB like Candidatus *Nitrosoglobus* have been enriched but not isolated in pure culture (39, 40). This hampers precise SynCom assembly. Single-cell Raman spectroscopy (SCRS) is a label-free, nondestructive technique that provides a biochemical fingerprint of an individual cell based on its intrinsic molecular vibrations (41). This provides deep insights into the physiological state and function of individual cells and is a forefront technique in single-cell metabolism research (42, 43). SCRS can identify microbial individuals with specific functions at the single-cell resolution level. Coupled with technologies like optical tweezers, it can further isolate these functional individuals. This method avoids the tedious operations and high costs associated with selective enrichment, significantly improving the speed, accuracy, and diversity of obtaining pure cultures of functional microorganisms (44).

Traditional approaches for obtaining such functional isolates rely on selective enrichment culturing, which is time-consuming, especially for slow-growing autotrophs such as AOB, which may require weeks to months, and often yields mixed cultures rather than pure isolates. For instance, Yuan et al. successfully cultivated a functional microbial community capable of stable NH_4_^+^ oxidation under acidic conditions (pH <5.0) by maintaining activated sludge in acidic wastewater for over 400 days (39, 40). The main acid-tolerant AOB identified was Candidatus *Nitrosoglobus*. Alternative techniques such as fluorescence-activated cell sorting require fluorescent labeling, which can perturb cellular functions and is not universally applicable to obtain active cells (42). Microfluidic droplet cultivation, while promising, typically requires prior knowledge of growth conditions and is less effective for unculturable or poorly characterized strains (45). In contrast, SCRS is a label-free, nondestructive technique that provides a biochemical fingerprint of an individual cell based on its intrinsic molecular vibrations (43). Specific Raman bands, such as those corresponding to cytochromes (∼750, 1,128, and 1,585 cm^−1^), lipids (∼2,850 cm^−1^ for CH_2_ stretch), nucleic acids (∼785 cm^−1^ for O-P-O backbone), and stress-related molecules (e.g., the OH stretch at ∼3,400 cm^−1^), can serve as intrinsic markers for cell identity, metabolic activity, and physiological state (46, 47). SCRS, thus, enables the identification of microbial individuals with specific functions at single-cell resolution without the need for prolonged enrichment.

**Fig 2:**
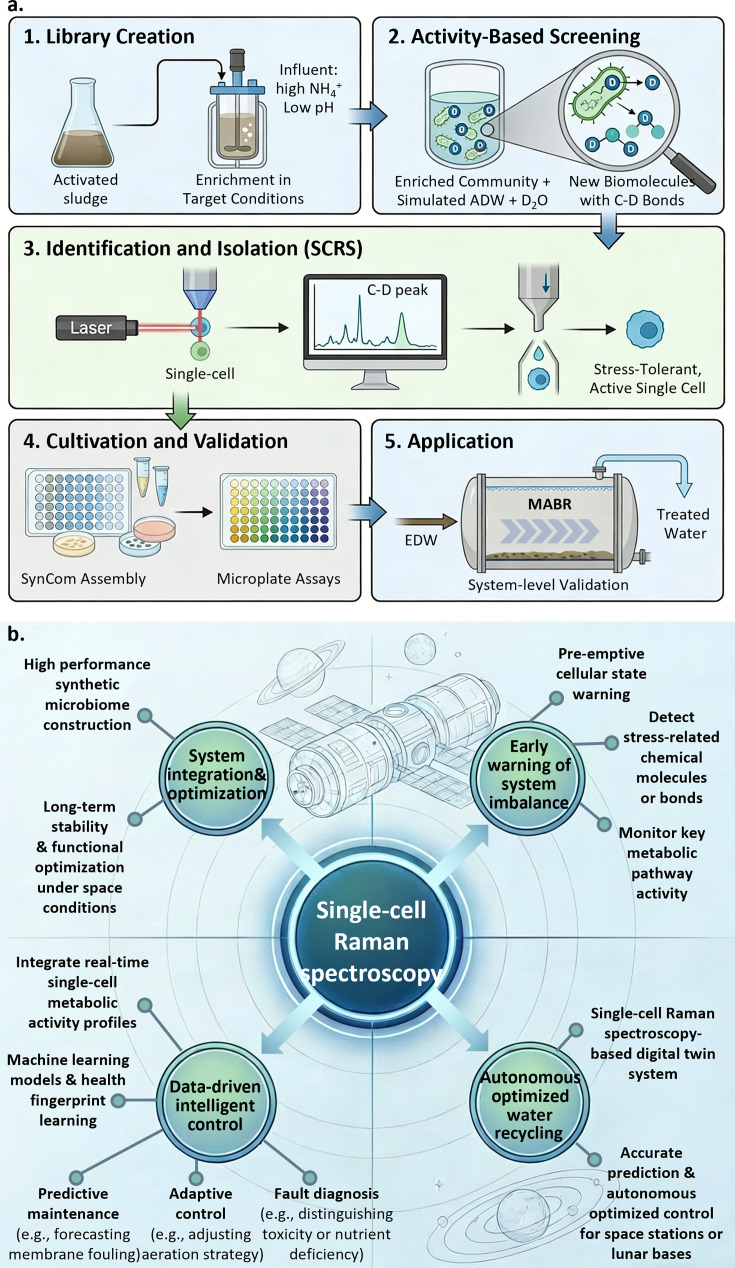
(**a**) A theoretical workflow for building stress-tolerant SynCom for EDW treatment with the assistance of SCRS technology. (**b**) Optimizing microbial-driven EDW treatment systems with the assistance of SCRS technology (assisted by Nano Banana Pro).

When coupled with stable isotope probing (e.g., using deuterium oxide [D_2_O]), single-cell Raman spectroscopy (SCRS) transforms into a powerful activity sensor (48). Active microorganisms incorporate deuterium into newly synthesized biomolecules, generating a distinct C-D Raman peak shift. This D_2_O-SCRS approach allows for the direct identification and sorting of metabolically active cells under specific environmental conditions, such as the stressful extraterrestrial domestic wastewater milieu. We propose a theoretical workflow for constructing a stress-tolerant SynCom, for EDW treatment begins with enriching a diverse inoculum under target stresses (Fig. 2a). The enriched community is then exposed to simulated EDW conditions with D_2_O. Using Raman-activated cell sorting (RACS), targeted individual cells (AOB and denitrifier) exhibiting strong C-D signals, indicating stress tolerance and activity, are isolated and cultivated into pure strains. These strains are assembled into candidate SynComs and validated for SCNR in microplate assays. Finally, the optimal consortium is inoculated into an MABR for system-level testing. This SCRS-guided strategy can enable the targeted isolation of functional strains to build a stable, NOB-free SynCom.

Importantly, SCRS can detect not only the presence of metabolic activity (via C-D peak) but also subtle shifts in cellular stress status. For instance, dose-dependent decreases in carotenoid-related Raman peaks (e.g., 1,155, 1,185, and 1,218 cm^−1^) and increases in the phenylpropanoid peak (1,632 cm^−1^) were, respectively, correlated with cadmium- and arsenic-induced oxidative stress and defense responses in rice (49). Similarly, a decline in the intensity of cytochrome bands indicates impaired electron transport chain function, providing an early warning of toxicity before growth cessation or cell death occurs (46). These spectral biomarkers form the basis for a rapid, culture-independent assessment of strain tolerance to EDW-specific stressors.

## SCRS-POWERED INTEGRATION AND OPTIMIZATION OF MICROBIAL-DRIVEN EDW TREATMENT SYSTEMS

The successful integration of microbial technologies into space life support systems requires not only the construction of high-performance synthetic consortia but also ensuring their long-term stability and functional optimization under unique space conditions. SCRS technology can be a pivotal tool in this system integration and optimization process, serving as a critical bridge from microscopic mechanistic understanding to macroscopic system control.

Deep-space missions demand systems with high autonomy and reliability. SCRS technology is capable of elevating microbial-driven EDW treatment operation management from post-facto water quality monitoring to pre-emptive cellular state warning (Fig. 2b). Before conventional water quality parameters (e.g., effluent ammonium) deteriorate, environmental stresses (e.g., shock loading and pH fluctuation) first manifest in the cellular physiology of functional microorganisms. SCRS can sensitively detect changes in Raman signatures of stress-related molecules (e.g., OH radicals and unsaturated fatty acid ratios) or decreased activity of key metabolic pathways (via D_2_O uptake rates), issuing an early warning prior to system functional failure. For example, a decrease in the C-D band intensity (reflecting reduced D_2_O uptake) in the AOB population following a pH drop could potentially be detected within hours, well before any measurable change in effluent ammonium concentration. Concurrently, an increase in the OH stretch band might serve as an early indicator of oxidative stress, while a shift in the lipid unsaturation ratio could signal membrane adaptation. By feeding such time-resolved, single-cell spectral data, alongside conventional parameters (e.g., dissolved oxygen, pH, and influent loading rates) and operational logs into a machine learning framework, a predictive model could be trained to recognize the baseline spectral fingerprint of the SynCom under varying conditions. This model could then issue pre-emptive warnings and recommend corrective actions, like adjusting aeration intensity or pH setpoint, before system performance degrades.

Real-time single-cell metabolic activity profiles from SCRS can be combined with macro-scale EDW treatment system operational parameters, online water quality sensor data, and operational history. These combined data sets can then be used to train machine learning models. These models can learn the health fingerprint of the microbial community under various conditions, enabling predictive maintenance (e.g., forecasting membrane fouling trends), adaptive control (e.g., automatically adjusting the aeration strategy in response to changing influent loads to maintain the SCNR pathway), and fault diagnosis (e.g., distinguishing between performance decline due to toxicity inhibition or nutrient deficiency). Ultimately, an SCRS-based digital twin system can be constructed to achieve accurate prediction and autonomous optimized control of water recycling processes on space stations or lunar bases. In summary, SCRS is not only a powerful screening tool for constructing synthetic consortia but also a core enabling technology for ensuring their stable colonization, functional expansion, and intelligent operation within MABRs in space. It deepens the dimension of system monitoring to the level of cellular metabolism, transforming space-based biological water treatment from an experience-dependent black box into a transparent, real-time observable, predictively controllable, and intelligently coupled resource regeneration hub within CELSS. This provides a revolutionary technological solution for life support assurance in long-term human space exploration missions.

Beyond space applications, the technologies and principles discussed herein, particularly the bottom-up construction of stable, stress-tolerant synthetic microbial consortia and the integration of SCRS for real-time, single-cell monitoring, hold significant promise for other extreme scenarios that demand self-sustaining and low-consumption wastewater treatment. Remote communities, rural areas, disaster-relief camps, and decentralized systems often face remarkably similar constraints. These include limited access to external consumables (e.g., chemicals for pH adjustment or external carbon sources), energy scarcity, and the need for robust, low-maintenance operation with minimal human intervention. In these settings, the same synthetic SCNR microbiome and Raman-based early-warning systems developed for extraterrestrial habitats could enable reliable, circular water recycling without frequent resupply. Thus, while extraterrestrial habitats represent the most stringent testbed for closed-loop water recycling, the solutions engineered under such extreme conditions are directly translatable to addressing pressing water challenges on Earth. We hope this review inspires cross-disciplinary efforts that leverage space research as a driver for innovation in terrestrial water sustainability.
